# Early innate responses to pathogens: pattern recognition by unconventional human T-cells

**DOI:** 10.1016/j.coi.2015.06.002

**Published:** 2015-10

**Authors:** Anna Rita Liuzzi, James E McLaren, David A Price, Matthias Eberl

**Affiliations:** 1 Division of Infection and Immunity, School of Medicine, Cardiff University, Cardiff CF14 4XN, UK; 2 Human Immunology Section, Vaccine Research Center, National Institute of Allergy and Infectious Diseases, National Institutes of Health, Bethesda, MD 20892, USA

## Abstract

Although typically viewed as a feature of innate immune responses, microbial pattern recognition is increasingly acknowledged as a function of particular cells nominally categorized within the adaptive immune system. Groundbreaking research over the past three years has shown how unconventional human T-cells carrying invariant or semi-invariant TCRs that are not restricted by classical MHC molecules sense microbial compounds via entirely novel antigen presenting pathways. This review will focus on the innate-like recognition of non-self metabolites by Vγ9/Vδ2 T-cells, mucosal-associated invariant T (MAIT) cells and germline-encoded mycolyl-reactive (GEM) T-cells, with an emphasis on early immune responses in acute infection.

## Highlights

• Vγ9/Vδ2 T-cells, MAIT cells and GEM T-cells sense distinct microbial patterns.
• Pattern recognition occurs via biased TCRs and non-polymorphic presenting molecules.
• Presenting molecules include MHC-like (MR1, CD1b) and non-MHC-like (BTN3) proteins.

**Current Opinion in Immunology** 2015, **36**:31–37

This review comes from a themed issue on **Host pathogens**

Edited by **Peter A Barry** and **Guido Silvestri**

For a complete overview see the Issue and the Editorial

Available online 13th July 2015

**http://dx.doi.org/10.1016/j.coi.2015.06.002**

0952-7915/© 2015 The Authors. Published by Elsevier Ltd. This is an open access article under the CC BY license (http://creativecommons.org/licenses/by/4.0/).

## Introduction

The human body is constantly exposed to a vast array of microorganisms through contact with environmental species and interactions with commensals, opportunists and pathogens. This microbial bombardment exerts a perpetual evolutionary pressure on the immune system to identify and eliminate potentially dangerous agents. Microbes express a plethora of pathogen-associated molecular patterns that engage with various components of the human immune system, triggering rapid and distinct responses as a first-line defense against specific groups of organisms. The innate recognition of such patterns ultimately induces unique clusters of immune and tissue-related biomarkers that coalesce as pathogen-specific ‘immune fingerprints’ [1•, 2], with widespread implications for point-of-care diagnosis of acute infection.

In the adaptive immune system, somatic recombination of V(D)J gene segments and junctional modifications generate a diverse repertoire of clonotypically expressed TCRs, enabling antigenic peptide-specific T-cell responses restricted by MHC class I and class II molecules. Although such genomic rearrangements occur in all T-cells, ‘unconventional’ populations characterized by semi-invariant, invariant or even germline-encoded TCRs are universally present and serve to recognize alternative antigens that are not restricted by classical MHC molecules. Research over the past three years has exposed how unconventional T-cells detect pathogens by sensing microbial, non-peptidic compounds via entirely novel antigen presenting pathways. High throughput sequencing approaches have also hinted at the existence of further unconventional T-cell subsets [3]. This review will focus primarily on the innate-like recognition of non-self metabolites by human Vγ9/Vδ2 T-cells, mucosal-associated invariant T (MAIT) cells and germline-encoded mycolyl-reactive (GEM) T-cells. The roles of other unconventional T-cells and iNKT cells in tissue homeostasis, stress surveillance and autoimmunity are well described elsewhere [4, 5, 6].

## Unconventional T-cells: Not based on or conforming to what is generally done or believed (Oxford Dictionary)

**Figure 1:**
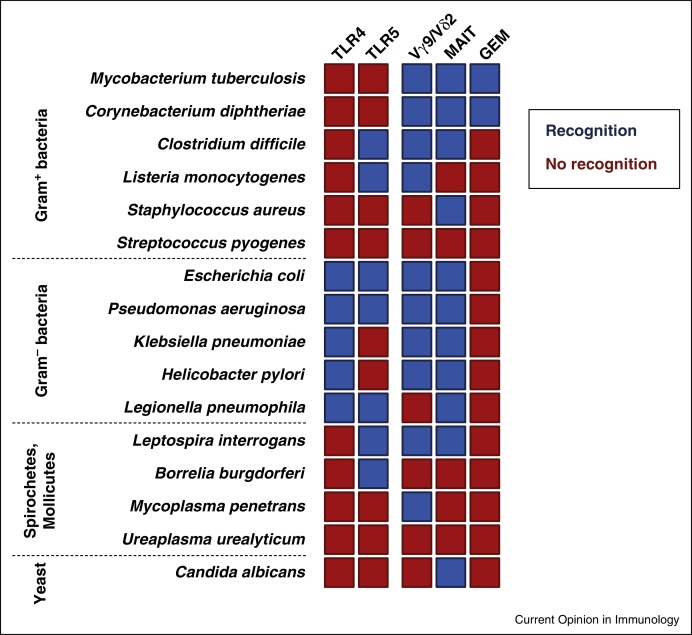
Innate sensing of microbial pathogens by Toll-like receptors and unconventional T-cell receptors. Pattern recognition of clinically relevant microbial pathogens via TLR4 and TLR5, and TCRs expressed by Vγ9/Vδ2 T-cells, MAIT cells and GEM T-cells. Blue symbols, recognition; red symbols, no recognition.

Given the energetic costs of somatic recombination and thymic selection (largely unproven for unconventional T-cells), innate-like recognition by certain αβ and γδ T-cells must confer a crucial evolutionary advantage. In this respect, Vγ9/Vδ2 T-cells, MAIT cells and other unconventional T-cells effectively bridge the innate and adaptive immune systems by orchestrating acute inflammatory responses and driving the generation of antigen-presenting cells [7•, 8, 9]. Akin to the discrimination between ‘self’ and ‘non-self’ via TLR4-mediated recognition of lipopolysaccharide (LPS), a cell wall constituent of Gram-negative bacteria, and TLR5-mediated recognition of flagellin, a component of bacterial flagella, the metabolic pathways targeted by Vγ9/Vδ2 T-cells, MAIT cells and GEM T-cells incorporate key structures that allow the body to sense a wide range of potentially harmful microorganisms and trigger an inflammatory response aimed at effective pathogen control (Figure 1). These biochemical determinants are absent from human cells and include ligands derived from the non-mevalonate pathway, which generates the building blocks of all higher isoprenoids in most Gram-negative bacteria and many Gram-positive species (as well as the protozoa *Plasmodium falciparum* and *Toxoplasma gondii*) [10], components of the riboflavin pathway, which yields vitamin B2 in the vast majority of bacteria as well as yeasts and fungi [11], and certain long-chain fatty acids (mycolic acids) found exclusively in the cell wall of mycobacteria and coryneform bacteria [12^••^]. Similar principles govern the recognition of microbial α-linked glycolipids by iNKT cells [5, 6]. Many unconventional T-cells also respond to cytokines such as IL-1β, IL-12, IL-18 and IL-23 in a TCR-independent manner, and may therefore act similarly to NK cells and other innate lymphoid cells [13, 14, 15].

To facilitate innate sensing of microbial pathogens, unconventional human T-cells are thought to undergo extrathymic and presumably antigen-driven expansion in the periphery, consistent with a predominant central or effector memory phenotype and the capacity to mount rapid responses. Unconventional T-cells also localize frequently to specific tissues and may therefore play a role in local immune surveillance. Intriguingly, human Vγ9/Vδ2 T-cell and MAIT cell numbers increase in peripheral blood after birth and subsequently decline in older individuals; they are also more prevalent in women [16, 17]. These observations could reflect age-related and gender-dependent exposure to environmental, commensal and/or pathogenic species. However, recent studies show that both Vγ9/Vδ2 T-cells and MAIT cells acquire their anti-microbial responsiveness during fetal development, prior to contact with environmental microbes and commensal microflora [18•, 19•]. Of note, an age-related decline associated with changes in peripheral subset composition has also been reported for iNKT cells [20].

## Vγ9/Vδ2 T-cells: Antigen presentation without presentation of an antigen?

**Figure 2:**
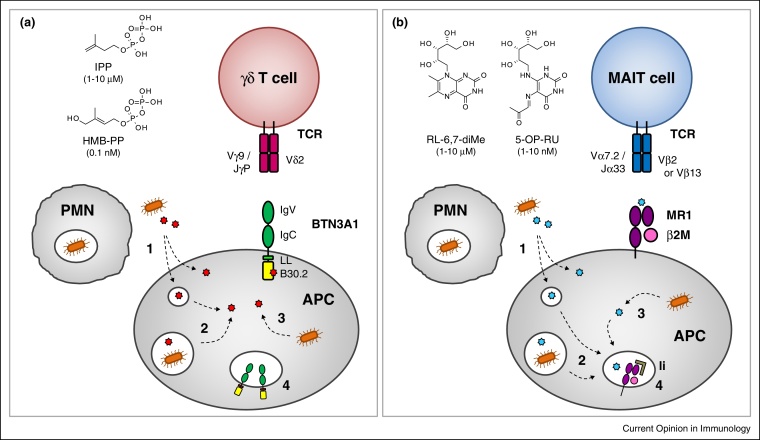
Recognition of microbial metabolites by unconventional T-cells. **(a)** ‘Presentation’ of HMB-PP to the Vγ9/Vδ2 TCR in a BTN3-dependent manner: *1*, Uptake of soluble HMB-PP released by extracellular bacteria, phagocytes or infected cells, via endocytosis and/or active/passive transport across the cell membrane (e.g. *E. coli*); *2*, Transport of HMB-PP from endocytic vesicles across the membrane after phagocytosis or infection (e.g. *Mycobacterium tuberculosis*); *3*, Release of HMB-PP into the cytosol by intracellular pathogens (e.g. *Salmonella* spp.); *4*, Putative intracellular loading compartment for BTN3. High affinity ligand: HMB-PP, (*E*)-4-hydroxy-3-methyl-but-2-enyl pyrophosphate; low affinity ligand: IPP, isopentenyl pyrophosphate. Note that HMB-PP is a microbial metabolite, whereas IPP is present in all prokaryotic and eukaryotic cells. **(b)** Presentation of vitamin B2 metabolites to the MAIT TCR by MR1: *1*, Uptake of soluble vitamin B2 metabolites released by extracellular bacteria, phagocytes or infected cells; *2*, Shuttling of vitamin B2 metabolites to late endosomes; *3*, Release of vitamin B2 metabolites into the cytosol; *4*, MR1 loading compartment. High affinity ligand: 5-OP-RU, 5-(2-oxopropylideneamino)-6-d-ribitylaminouracil; low affinity ligand: RL-6,7-diMe, 6,7-dimethyl-8-d-ribityllumazine. APC, antigen-presenting cell; PMN, polymorphonuclear cell; IgV, immunoglobulin-like V ectodomain; IgC, immunoglobulin-like C ectodomain; LL, di-leucine motif; β2M, β_2_-microglobulin; li, MHC class II-associated invariant chain (CD74).

Peripheral blood Vγ9/Vδ2 T-cells carrying a *TRGV9*/*TRGJP*-encoded TCRγ chain normally constitute 1–5% of the circulating T-cell population in humans but can increase in frequency to >50% during microbial infections. Despite their prevalence in blood, these cells mobilize rapidly to mucosal surfaces, where they may confer protection against tissue-localized infections [21]. Vγ9/Vδ2 T-cells display a striking responsiveness to bacterial species capable of producing the isoprenoid precursor (*E*)-4-hydroxy-3-methyl-but-2-enyl pyrophosphate (HMB-PP) *in vitro* and *in vivo* [9, 22] (Figure 2). In patients infected with a range of pathogens, HMB-PP-producing organisms are associated with higher Vγ9/Vδ2 T-cell frequencies than HMB-PP-deficient species. This appears to be true both for local responses at the site of infection, as demonstrated in patients with acute bacterial peritonitis [1•, 10], and for systemic responses during acute sepsis [7^•^]. These clinical observations are backed up by studies in macaques, where an HMB-PP-deficient vaccine strain of *Listeria monocytogenes* elicited significantly reduced pulmonary and systemic Vγ9/Vδ2 T-cell responses compared with the HMB-PP-producing parental strain [23^•^]. Similarly, an HMB-PP-overproducing vaccine strain of *Salmonella enterica* serovar Typhimurium stimulated prolonged Vγ9/Vδ2 T-cell expansions in rhesus monkeys, while the avirulent parental strain was less efficient in this respect [24].

The unique responsiveness of Vγ9/Vδ2 T-cells to HMB-PP remains enigmatic as it appears to embody the only case where an antigen is not actually presented to the TCR but instead binds intracellularly to an innate sensor (butyrophilin 3; BTN3/CD277) (Figure 2). Although Vγ9/Vδ2 T-cells are generally portrayed as a population unique to primates and absent in rodents, immunogenetic studies point to a co-evolution of the Vγ9 and Vδ2 genes together with *BTN3* in other placental mammals such as alpacas [25]. However, functional proof for the presence of HMB-PP-specific and BTN3-dependent Vγ9/Vδ2 T-cells in these species is still lacking. Following the pioneering discovery of BTN3 as a restriction element for human Vγ9/Vδ2 T-cell responses [26, 27••] and the observation that anti-BTN3 agonist antibodies and soluble phosphoantigens induce identical signaling pathways [28], at least four independent studies have provided evidence for direct binding of HMB-PP to a positively charged pocket in the cytosolic B30.2 (PRYSPRY) domain of BTN3A1 [29••, 30, 31•, 32•]. These findings contradict an alternative model proposing that HMB-PP binds to the extracellular IgV domain of BTN3A1 and is therefore a truly presented antigen [27^••^]. Despite this convergence of experimental data, it remains entirely unclear how the Vγ9/Vδ2 TCR actually recognizes the BTN3A1/HMB-PP complex. Inside the cell, HMB-PP sensing might be accompanied by interaction partners such as periplakin, which binds a di-leucine motif located proximal to the cytoplasmic B30.2 domain of BTN3A1 [32^•^]. These intracellular events may subsequently propagate across the cell membrane and induce conformational changes on the cell surface [33], possibly in combination with co-factors or clustering effects that enable recognition by Vγ9/Vδ2 TCRs. Nevertheless, it is challenging to reconcile how the detection of a seemingly ubiquitous and non-polymorphic molecule such as BTN3A1 is facilitated through a rearranged TCR.

## MAIT cells: Unconventional pathogen-sensing through conventional TCR diversity?

MAIT cells are innate-like T-cells that populate mucosal tissues such as the intestine and lung, comprising in addition up to 10% of the circulating CD8^+^ αβ T-cell compartment and as many as half of all T-cells present in the liver [34]. They share phenotypic similarities with iNKT cells, express IL-12Rβ2 and IL-18Rα alongside high levels of CD161 [14, 35], and localize to sites of infection via chemokine receptors such as CCR2, CCR6 and CXCR6 [35, 36]. Unlike conventional αβ T-cells, MAIT cells possess thymic effector functionality despite a naïve phenotype [34] and are selected by hematopoietic cells [37]. They subsequently expand in the periphery as antigen-experienced, effector memory T-cells upon microbial exposure [36, 37]. Of note, the presence of an intact commensal flora and expression of the non-polymorphic MHC-related protein 1 (MR1) by B-cells are both essential for this peripheral expansion, whereas macrophages and dendritic cells are dispensable [37]. Recent data have also revealed an essential role for STAT3 signaling downstream of IL-21R and IL-23R in controlling human MAIT cell numbers [38^•^].

Pathogen recognition by human MAIT cells is driven by a semi-invariant MR1-restricted TCR that typically incorporates a TRAV1-2/TRAJ33 (Vα7.2-Jα33) TCRα chain paired predominantly with a TRBV20-1 (Vβ2) or TRBV6 (Vβ13) TCRβ chain. Infrequent usage of other *TRAJ* and *TRBV* gene segments has also been described [3, 39]. Unlike MHC class I-restricted epitopes, MAIT cell ligand presentation by MR1 is independent of proteasomal degradation and the transporter associated with antigen processing (TAP), but requires the MHC class II chaperones invariant chain (li) and HLA-DM [40]. After initial observations that both human and murine MAIT cells respond to species such as enterobacteria, staphylococci and mycobacteria, but not to streptococci [7•, 36], key mechanistic advances have shown that MAIT TCRs recognize ligands derived from microbial vitamin B2 metabolism [11, 41, 42••] (Figure 2). Recent analyses have also revealed that the MAIT cell repertoire is more diverse than initially thought [43, 44•], which may allow these cells to discriminate between different microbial pathogens via TCR-dependent ‘sensing’ of distinct MR1-bound ligands [39, 44•]. These findings suggest the existence of other, as yet undiscovered, microbial antigens within the MAIT cell recognition spectrum, a possibility consistent with structural interpretations of MR1 ligand promiscuity [41, 45, 46, 47]. However, a recent study in mice has challenged this idea of ligand discrimination via the TCRβ chain [48], which may point to species-specific differences between human and murine MAIT cells.

Patients with severe sepsis display an early decrease in circulating MAIT cells compared with healthy controls and uninfected critically ill patients [49^•^]. In particular, non-streptococcal bacterial infection was identified as an independent determinant of peripheral MAIT cell depletion, suggesting recruitment to the site of infection in response to pathogens with an intact riboflavin pathway [36, 50, 51]. In HIV-1 infection, circulating Vα7.2^+^ CD161^+^ T-cells are depleted and fail to recover with antiretroviral therapy [52, 53]. This may indicate a progressive translocation of MAIT cells to peripheral tissues, down-regulation of CD161, functional exhaustion and/or activation-induced apoptosis. In a number of autoimmune and metabolic disorders, MAIT cells typically display similarly decreased levels in peripheral blood [54, 55, 56], possibly as a result of low-grade inflammation and alterations of the microbiota.

## Other pathogen-specific unconventional T-cells: GEM T-cells and beyond

The MHC class I-related molecule CD1b was found almost 20 years ago to present bacterial glycolipids such as glucose monomycolate (GMM), yet the identity and specificity of CD1b-restricted T-cells has remained elusive until recently [57]. Mycolic acids (MAs) are the predominant cell wall lipids in *Mycobacterium tuberculosis* and represent a major virulence factor for this pathogen. Rare MA-specific T-cells are detectable in tuberculosis patients at diagnosis but virtually absent in non-infected BCG-vaccinated individuals [58]. These T-cells are CD1b-restricted, exhibit both central and effector memory phenotypes, produce IFN-γ and IL-2 upon stimulation, and appear to localize preferentially at the site of infection. The availability of CD1b tetramers allowed direct visualization of MA-specific T-cells, which were estimated to comprise approximately 0.01% of all circulating T-cells [59]. These advances eventually led to the description of CD1b-restricted T-cells as Vα7.2^+^ CD4^+^ germline-encoded mycolyl-reactive (GEM) T-cells, which carry an invariant TRAV1-2/TRAJ9 TCRα chain [12^••^]. MA-specific T-cells were also shown to decline after successful treatment and therefore appear to correlate with pathogen burden [58], emphasizing the potential importance of these unconventional T-cells as novel diagnostic and prognostic biomarkers of tuberculosis.

As mycolic acids are a hallmark of all Corynebacteriales, it is tempting to speculate that MA-specific T-cells may also sense infections caused by bacteria such as *Corynebacterium* spp. and *Nocardia* spp. (Figure 1). Of note, a second population of GMM-specific T-cells has been identified recently. These cells exhibit lower avidities for CD1b tetramers and, in contrast to GEM T-cells, express TCRs with a marked preference for the *TRAV17* and *TRBV4-1* genes [60]. High throughput sequencing of TRAV1-2^+^ TCRα chains further suggests that we are only seeing the tip of the iceberg with regard to our knowledge of unconventional T-cell populations [3]. It therefore seems likely that many exciting discoveries will ensue in this hybrid field.

## Conclusions and future directions

The last three years have witnessed major advances in our understanding of unconventional T-cell subsets, in part due to the skillful application of cutting-edge experimental techniques to well-defined patient cohorts. Future research can now build on this foundation to define the true extent of these T-cell populations and define the mechanisms that underlie microbial pattern recognition within the adaptive immune system. Many questions remain in this regard. Precisely how do unconventional TCRs interact with non-polymorphic presenting molecules? Are specific gene segments within the TCR locus conserved for this purpose? Does the process of somatic recombination serve to diversify bound ligand recognition? Do unconventional T-cells undergo positive selection in the thymus and does this process involve the engagement of endogenous ligands? What are the molecular processes involved in antigen uptake and intracellular trafficking that allow the presentation of microbial metabolites?

Key pieces of the puzzle are also missing at the functional level. How do unconventional T-cells migrate to and from sites of infection? Do they persist as tissue-resident memory-like cells after pathogen clearance? What is the role of the commensal microbiota? Why do most unconventional T-cells possess a memory phenotype from early life? What mechanisms underlie the pronounced age and gender bias? Are there implications for homeostasis and susceptibility to infections, autoimmunity and malignancy? How do accessory molecules such as CD4, CD8, CD161 and NKG2D contribute in this setting?

It is becoming increasingly clear that unconventional T-cells play a pivotal role in the orchestration of early inflammatory responses. In parallel, emerging mechanistic insights have started to unlock the secrets of innate-like recognition encoded by specific portions of the TCR repertoire. The highly constrained genetic and microbial elements inherent within each of these various systems potentially offer unique molecular targets for the development of novel and universally applicable diagnostics, vaccines and immunotherapeutics. The overarching question is therefore, as always, a humanitarian one. How can we best harness the unique attributes of unconventional T-cells to combat the infectious and malignant plagues of our times?

## Conflict of interest statement

The authors declare no competing financial interests.

## References and recommended reading

Papers of particular interest, published within the period of review, have been highlighted as:

• of special interest
•• of outstanding interest
